# Comparison of Endodontic Biomaterials as Apical Barriers in Simulated Open Apices

**DOI:** 10.5402/2012/359873

**Published:** 2012-06-27

**Authors:** Mamak Adel, Moradi Majd Nima, Shiva Shivaie Kojoori, Hooryeh Norooz Oliaie, Neda Naghavi, Saeed Asgary

**Affiliations:** ^1^Dental Carries Research Center, School of Dentistry, Qazvin University of Medical Sciences, Qazvin 34157-59811, Iran; ^2^Dental Research Center, Shahid Beheshti University of Medical Sciences, Tehran 1983963113, Iran; ^3^Dental Research Center, School of Dentistry, Mashhad University of Medical Sciences, Mashad 91375-3316, Iran; ^4^Iranian Center for Endodontic Research, Dental Research Center, Shahid Beheshti University of Medical Sciences, Tehran 1983963113, Iran

## Abstract

*Objectives.* To evaluate the effect of apical foramen diameter and apical barrier thickness on the sealing ability of mineral trioxide aggregate (MTA) and calcium enriched mixture (CEM) plugs in open apices. *Materials and Methods.* The fluid filtration method was conducted on a total of 136 roots. Samples were randomly divided into two control (*n* = 8) and four experimental groups (*n* = 30). Apical foramen diameters measuring 1.1 and 1.7 mm were shaped for groups “1 and 3” and “2 and 4”, respectively. In groups 1 and 2 MTA plug and in groups 3 and 4 CEM plug was inserted. The groups were further divided into subgroups according to the thickness of the apical plugs (3- or 5-mm). Microleakage was measured at 1, 7, and 30 days. *Results.* Mixed ANOVA test showed that the microleakage in groups 1 and 3 as well as all 5-mm plug subgroups were significantly less than groups 2 and 4 (*P* < 0.05) and 3-mm subgroups (*P* < 0.05), respectively. Microleakage was significantly lower at 30th day (*P* < 0.05). *Conclusions.* Reducing canal diameter or increasing apical plug thickness and the time interval increases the sealing ability of apical barriers. Furthermore, in comparison to MTA, CEM plugs demonstrated superior sealing ability.

## 1. Introduction

Pulp necrosis in teeth with incomplete root structure can interrupt dentin formation and arrest root development. As a result, canal walls will be thin and fragile and apex of tooth remains open [1]. Therefore, root canal instrumentation is impaired and achievement of an adequate apical stop is impossible. In such cases, two techniques have been suggested [2]: the first involves removal of the necrotic tissue followed by debridement of the canal and placement of calcium hydroxide for multiple sessions to induce apical closure. The second technique commonly known as one-step apexification, an apical barrier is inserted to achieve an adequate apical stop. 

Apexification with calcium hydroxide has been successfully performed for a long time. However, this method requires patient compliance and multiple sessions, and due to the presence of thin roots or prolonged exposure of root dentin to calcium hydroxide, the tooth will be more susceptible to root fracture [3]. Therefore, one-step apexification procedure is becoming increasingly popular; different materials have been suggested as osteoconductive apical barriers in this method, for example, dentin chips, mineral trioxide aggregate (MTA) [4], and calcium enriched mixture (CEM) cement [5, 6].

The sealing ability and thickness of MTA apical plug have been evaluated by several studies [7–11], and most demonstrated good sealing ability [7–11]. These articles assessed the sealing ability of several thicknesses of apical barriers by using marginal apical dye leakage technique [8, 9] or a bacterial leakage model [5, 7]. They showed improved sealing ability of MTA plugs with greater thickness [10]. Furthermore, according to a prospective clinical study, MTA showed a high success rate when used as root-end filling material [12]. MTA is an excellent biocompatible material [10, 13], with a few drawbacks such as a long setting time, high cost, and potential for discoloration [3]. It does not have good handling characteristics [14] and its antibacterial properties are unpredictable [15]. 

To overcome these problems a new biomaterial was developed called calcium enriched mixture (CEM) cement using different calcium compounds [16]. CEM cement has a good sealing ability when it used as root-end filling material [17] and has shown favorable biologic response as a pulp capping agent [18]. It also has acceptable physical properties; its setting time is less than 1 hour and has greater flow and less film thickness than MTA [18]. In addition, it is capable of inducing hydroxyapatite formation over itself in normal saline solution [19].

In previous studies the effect of diameter of apical foramen on the seal ability of apical barriers was not assessed; therefore, the objectives of this study were (1) to investigate and compare the apical microleakage of MTA plug and CEM plug, using the fluid filtration method, and (2) to evaluate the effect of diameter of apical foramen and thickness of these biomaterials on their seal ability.

## 2. Materials and Methods

This study was approved by the Ethics Committees of Dental Research Center of Shahid Beheshti Medical University, Tehran, Iran, and Qazvin Medical University, Qazvin, Iran. A total of 136 maxillary anterior teeth were selected. Selected tooth samples all had single-rooted intact roots with completely formed apices. All teeth were examined in a stereomicroscope (MBC-2, Russia) under × 16 magnification, and teeth with cracks, fracture, canal calcification, and external root resorption were excluded. For disinfection, the specimens were stored in 5.25% sodium hypochlorite for an hour and then placed in normal saline before the experiment. 

In order to standardize all specimens to a length of 15 ± 1 mm, the crowns of the teeth were removed by sectioning with a diamond disc (Jota, Germany) attached to laboratorial handpiece. Subsequently, real root canal lengths were determined by manually inserting #15 K-files (Mani, Japan) into the canals, until the instrument tips were visible at the apical foramen. Working length was established 1.0 mm shorter than real root canal length. Apical instrumentation of roots was carried out with stainless steel files to a size of 40 K-file as a master apical file, subsequently canals were flared up to #80 with the step back technique. A K-type patency file size of #15 was used for the canal preparation phase, canals were irrigated with 1.0 mL of 2.5% sodium hypochlorite. After instrumentation was completed, 3.0 mL of 17% EDTA (Ariadent, Iran) were introduced and allowed to remain in the canals for 3 minutes. Next, a final flush with 1.0 mL of 2.5% sodium hypochlorite followed by 5.0 mL of normal saline was performed.

Following these steps, 3 mm of the apical roots were removed by sectioning with a diamond disk attached to laboratorial handpiece. Samples were randomly placed into four experimental groups (*n* = 30) and two negative and positive control groups (*n* = 8). The groups were further divided into subgroups of 15 teeth each, according to the thickness of the apical plugs (3 and 5 mm).

In group 1, apical foramen was enlarged by using Peeso drill up to #3 to simulate an open apex root canal, diameter = 1.1 mm. After this stage, diameter of apical foramens and the absence of any fracture or crack were confirmed by using a digital caliper (Mitutoyo Products Corp., Japan) and a stereomicroscope, respectively. Slices of sponge were soaked in PBS solution and teeth were placed in these slices to simulate periapical tissue. Thereafter, the roots were dried by paper point size of #80 (Ariadent, Iran), and MTA (Tooth-color ProRoot MTA, Dentsply, USA) was prepared according to the manufacturer's instructions, and carried with a MTA carrier (Maillefer, Swiss). The MTA was condensed up to the apical end with aid of plugger #3 and #4 (Maillefer, Swiss) with a rubber stop positioned 3 and 5 mm shorter than the root canal length, the excess material was removed for fabrication of 3 and 5 mm thick apical plugs, respectively. After apical plug fabrication, a moistened paper point was placed within the canals and then the density and thickness of apical plugs were confirmed by periapical radiography.

In group 2, apical foramen was enlarged by using Peeso drills up to #6 to simulate an open apex root canal, diameter = 1.7 mm. MTA plug was created identical to group 1 to seal apical portion of these canals.

In group 3 and 4, samples were prepared the same as group 1 and 2, respectively, but instead of MTA, CEM cement (BioniqueDent, Iran) plug was used to seal apical portion of root canals.

In positive control group, no apical plug was fabricated, in negative control group, 5 mm MTA apical plug was fabricated and after incubation root surfaces were completely covered by three coats of nail polish. 

All teeth were stored at 37°C in 95% humidity for 24 hours. After this period, the external root surfaces of the specimens in the experimental and the positive control groups were completely covered by three coats of nail polish and one layer of cyanoacrylate glue (Interlock, Japan), except for an area of 2.0 mm around the root apex. Root surfaces of the specimens in the negative control group were completely covered. 

Leakage was evaluated by the fluid filtration technique employing a pressure equivalent to 0.5 atmosphere, as described by Wu and Wesselink [20]. Four measurements were recorded for each tooth at 2-minute intervals over a period of 8 minutes at 1, 7, and 30 days. The amount of leakage was expressed as *μ*L/min/cm H_2_O. Kolmogorov-Smirnov test was used in order to determine normality of dispersal distribution of parameters; thereafter, results were analyzed by the mixed ANOVA (three-way and repeated measure ANOVA). The significance level was set at 5% for all tests.

## 3. Results

The positive control group showed the maximum amount of leakage, while the negative control group did not show any apical leakage. All samples in the experimental groups demonstrated variable amounts of apical leakage except for the CEM cement 5 mm thick plug at 30 days which had no apical leakage.  Table 1 shows microleakage values for different groups at several intervals according to the materials, plug thickness, and diameter of the apical foramen. A statistical significant difference (*P* < 0.05) was observed between the various amounts of microleakage in teeth with 1.1 mm apical foramen diameter and teeth with 1.7 mm apical foramen diameter, with both biomaterials and thickness of plugs, the 1.1 mm apical foramen diameter had less apical leakage compared to the larger diameter.

At 1, 7, and 30 days following apical plug fabrication 5 mm thickness of the two apical plugs showed less leakage than 3 mm thick root-end materials at all the three intervals (*P* < 0.05). Almost all the MTA groups had significantly greater microleakage compared to CEM (*P* < 0.05); however, in the first interval the 3 mm thick plug of CEM cement was showed the greatest amount of leakage (*P* < 0.05).

The degree of apical leakage following the 30 day was less than 1 day interval in all subgroups. Also, microleakage values had a significant two-way interaction with the thickness of apical plugs and a significant three-way interaction with the materials utilized and the apical foramen diameters, in several of the intervals. 

**Figure 1:**
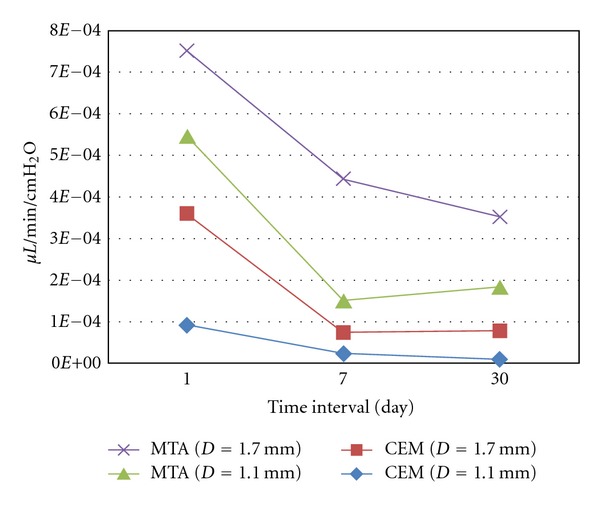
Leakage of MTA and CEM plugs in simulated open apices with various diameters.

Figure 1 shows the trend of microleakage in the root canals with the two tested biomaterials as well as two foramen diameters.

## 4. Discussion

Various methods have been suggested to determine the sealing ability of apical plugs, such as polymicrobial leakage, linear dye leakage, fluid filtration, diaphanization, radioisotope labeling, and the electrochemical method [21]. Dye penetration and bacterial leakage are more widely used [21]. The dye penetration method is qualitative in nature and also destructive, so longitudinal evaluation of microleakage is impossible. In addition, it was shown that alkaline materials cause methylene blue discoloration, which may lead to unreliable conclusions in dye penetration tests [22]. Bacterial studies are also qualitative and one passing bacterium through the filled root canal can indicate positive cultures [21]. Moreover, dry environment during the samples sterilization may cause crack or fracture in hydrophilic materials used as apical plugs. Another accepted method is fluid filtration, which was first introduced by Pashley's group in 1987 and modified by Wu et al. in 1993 for use in root canals [20, 21]. We employed this method; in this method, samples are not destroyed, so the longitudinal sealing ability can be assessed. Since very small volume can be recorded in this method, the results are accurate [20].

This study evaluated the sealing ability of MTA and CEM cement apical plugs, these biomaterials are commonly used in clinical practice [5, 23–25], where appropriate adaptation of the master gutta-percha cone is impossible. The goal of this apical barrier is to provide a tight seal [12]. In the present study, greater microleakage was observed in teeth with larger apical foramen diameters. This result seems perfectly reasonable. However, not many studies have demonstrated this empirically. Also in this study 5 mm thick plugs were more efficient for apical sealing than 3 mm thick plugs in all three intervals, regardless of the material utilized, which is in agreement with previous studies [5, 7, 9].

According to our results, apical microleakage was lowest at the 30-day interval, that is, an increasing period of time improved the seal. This is likely to be due to MTA and CEM apical plugs ability to induce hydroxyapatite formation in PBS solution, therefore creating a second seal and exhibiting less microleakage after 30 days [19].

There are a few studies that have assessed the association between time and apical plugs leakage. Gandolfi et al. [26] studied the apical sealing ability of two experimental retrograde root-filling cements and MTA using fluid filtration method. They reported reduced fluid flow rate over time. Also, Martin et al. [27] evaluated sealing properties of MTA orthograde apical plugs in an *in vitro* apexification model at 48 hours and after 4 weeks of immersion in PBS. They found that MTA apical plugs exhibited superior seal after 4 weeks. 

The present findings show that CEM apical plugs had less leakage than MTA apical plugs, regardless of thickness, apical foramen diameters, or the passage of time. The exact mechanism by which CEM biomaterial promotes sealing is currently unknown. This characteristic is likely to be the result of several properties such as physical properties (i.e., improved flow, good film thickness, and small-size particles) [18, 28] and/or chemical properties (i.e., calcium and phosphate ions release, hydroxyapatite formation, and similar surface composition to dentin) [19, 29, 30]. However, recent *ex vivo* and *in vivo* studies demonstrated that MTA and CEM cement present antimicrobial effect [31] as well as biocompatibility [12, 18, 32–41].

## 5. Conclusion

Within the limitations of the present *in vitro* study, it can be concluded that increasing the diameter of apical foramen or reduction of apical plugs thickness, significantly increases the apical microleakage of apical barriers. The lapse of time also improves sealability of endodontic biomaterials. Also in comparison to MTA, using the CEM plugs in simulated opened apex teeth had a better result. Further clinical studies are recommended to establish the results.

## Figures and Tables

**Table 1:** Mean (SD) leakage values for MTA and CEM plugs at three intervals according to the plug thickness (*T*) and canal diameter (*D*).

		Time intervals					
		1 day		7 days		30 days	
		*D* = 1.1 mm	*D* = 1.7 mm	*D* = 1.1 mm	*D* = 1.7 mm	*D* = 1.1 mm	*D* = 1.7 mm
CEM plug	*T* = 3 mm	1.43 (2.71)^∗^	4.66 (3.39)	3.30 (6.35)	5.39 (1.14)	1.78 (3.97)	1.12 (1.85)
	*T* = 5 mm	4.06 (9.69)	6.93 (1.31)	1.43 (3.88)	4.62 (9.96)	0.00 (0.00)	2.45 (5.44)
MTA plug	*T* = 3 mm	2.30 (1.71)	2.30 (1.62)	9.71 (1.34)	2.90 (6.07)	8.59 (8.34)	1.92 (4.77)
	*T* = 5 mm	1.42 (1.35)	1.80 (1.39)	5.69 (1.20)	2.94 (3.79)	1.26 (1.22)	1.44 (2.24)

*μL min^−1^ cm H_2_O^−1^ × 10^−4^.
